# Development of a survey on the activity participation of children with special needs in preschool inclusive education

**DOI:** 10.3389/fpsyg.2022.979677

**Published:** 2022-12-09

**Authors:** Li Xu, Zhiheng Xiong, Li Zeng

**Affiliations:** ^1^College of Educational Science, Nanjing Normal University of Special Education, Nanjing, China; ^2^School of Biological Science and Medical Engineering, Southeast University, Nanjing, China; ^3^College of Educational Science, Wenzhou University, Wenzhou, China

**Keywords:** preschool inclusive education, special children, participation, learning participation, teacher support

## Abstract

Participation refers to an individual’s psychological and behavioral participation in activities. The activity participation of children with disabilities is an important index to measure preschool inclusive education. This study explored the preparation of a questionnaire suitable for measuring the participation of children with disabilities in inclusive education environments, so as to provide an evaluation tool for researchers and teachers. The study included exploration of the four dimensions of learning participation, life participation, teacher support, and parental attitude of children with disabilities in inclusive education. The research involved kindergarten teachers with inclusive education experience in 10 provinces and 20 cities in China such as Jiangsu, Zhejiang, Anhui, Jilin, Inner Mongolia, Sichuan, Guizhou, Guangxi, Yunnan, and Xinjiang. Questionnaire is filled in the form of voluntary principle and online. The Cronbach’s α coefficient for the total dimension of the questionnaire was 0.950, with coefficients for each sub-dimension ranging from 0.916 to 0.981. The Mplus8.3 software was used to carry out confirmatory factor analysis on the four-factor model. The standardized load of the four sub-dimensions was between 0.88 and 0.95. The load reached the significant level of 0.01, the interpretation rate presents a medium and high level (the interpretation rate is greater than 70%), and the model fits well. Therefore, based on good reliability and validity, this questionnaire can be used to evaluate the activity participation of children with disabilities in preschool inclusive education.

## Introduction

Inclusion, in essence, is a sense of belonging and participation which gives full play to people’s potential in a diversified society (Odom et al., 2011). With the continuous advancement of practice, the quality of preschool inclusive education, especially the participation of children with disabilities in inclusive education, has attracted increasing attention. Participation reflects the individual’s psychological and behavioral participation in activities. The activity participation of children with disabilities in the field of inclusive education is an important indicator of the quality of preschool inclusive education. Communist Party of China (CPC) Central Committee and State Council issued *The Overall Plan for Deepening the Reform of Educational Evaluation in the New Era*, which clearly puts forward that the focus of kindergarten evaluation is to evaluate the scientific protection and education of kindergartens and to standardize their operation. All kinds of kindergartens should be included in the scope of quality evaluation, and relevant evaluation tools are urgently needed for kindergartens that accept children with disabilities and their inclusive education activities. As far as the current relevant research is concerned, it mainly focuses on the definition of children’s participation, the ladder of participation, the participation of children with special education needs in different types of activities in inclusive education, and the introduction of foreign preschool inclusive education evaluation tools. From the existing quality evaluation indicators of preschool inclusive education, the connotation of children with disabilities’ activity participation is not clearly defined, and it is difficult to see the evaluation tools of children with disabilities’ activity participation in the field of inclusive education. In order to further promote the high-quality development of preschool inclusive education and realize the equality of educational opportunities, this study focused on the actual participation process and input of children with disabilities in the inclusive education field and explored and formulated a questionnaire of participation degree of children with disabilities in preschool inclusive education.

### Policies and practices of pre-school inclusive education

“Inclusive education” is essentially the process of promoting children’s participation and is the concrete embodiment of educational equity and an important way to improve social inclusion. The basic purpose of the Convention on the Rights of Persons with Disabilities is to promote, protect and ensure the full and equal enjoyment of all human rights and fundamental freedoms for all persons with disabilities, and to promote respect for the inherent dignity of persons with disabilities. The guide to The United Nations’ *Convention on the Rights of Persons With Disabilities* states that “inclusive education not only provides the best educational environment but also helps to break barriers and challenge stereotypes (MacKay, 2006).” “The State party recognizes the right of education to persons with disabilities. In order to achieve this right without discrimination and equal opportunity, States parties should ensure that an inclusive education system and lifelong learning at all levels of education.” China is an active advocate, firm supporter and implementer of the Convention. Since its first implementation report in 2012, China has earnestly implemented the Convention and respected and protected the human rights of persons with disabilities. The concept of human rights advocated by the Convention has been promoted in the whole society. In 1990, *The Law of the People’s Republic of China on the Protection of Disabled People* clearly stated that the general early childhood education institutions should accept disabled children who can adapt to their lives. In 1994, *The Salamanca Declaration* formally proposed inclusive education. In 2006, *The International Convention on the Rights of Persons With Disabilities* stipulated that States parties should take all necessary measures to ensure that children with disabilities enjoy fully all human rights and fundamental freedoms on the basis of equality with other children. From an international perspective, various documents issued by the United States, Britain, Japan, and other countries emphasize the importance and specific guidance of pre-school inclusion education.

*The Act on Early Assistance for Disabled Children* is an early preschool inclusive education policy issued in 1968 in the United States, and was the first federal act to protect the rights and interests of children with disabilities. In 1972, *The Early Start Plan* required that the participation rate of children with developmental disabilities at no less than 10% of the total. *The Education Law for All Disabled Children* promulgated in 1975 stated that the government should provide free and appropriate public education for children with disabilities, and pre-school disabled children were included in the education system. *The Act of “No Child Left Behind “*in 2002 regulated the identification procedure for children with disabilities, and paid attention to the rehabilitation of children with disabilities and related research. In 2009, *The Joint Statement of Preschool Inclusive Education: The American Children with disabilities’s Committee Preschool Education Branch and the American Association for Early Childhood Education* (AIE) proposed that the characteristics of the inclusion of high-quality early education programs include access to opportunities, active participation, and systematic support.

*The Warnock Report* in 1978 made inclusive education the core of special education. In 1999, *The Guidance on A Sound Start-Up* proposed better pre-school education, game activities, and health services for children with disabilities. The *Charter of Special Education Needs Identification and Evaluation* is used to diagnose and evaluate special educational needs, and the targeted tasks are put forward.

In 1971, *Japan’s Basic Implementation Measures for Expanding and Improving The Education of the Future Educational Institutions* took pre-school inclusive education as an important link of the “special education expansion plan.” *The Implementation Outline of Pre-school Inclusion Education* in 1974 stipulated that children over 4 years old who were in a state of lack of care must enter kindergarten every day. *The Private Child Care Institution Revitalization Grant Act* 1975 provided subsidies for private kindergartens receiving pre-school children with disabilities with physical and mental disorders.

In China, the relevant policies and regulations on promoting pre-school inclusive education in various documents are increasingly clear and specific. Since 2009, the National Human Rights Action Plan has been formulated and implemented, clarifying the tasks and requirements for protecting the human rights of persons with disabilities, and has formulated and implemented three special national plans for developing the cause of disabled persons and protecting them during the 13th Five-Year Plan period and the 14th Five-Year Plan period.

The 14th Five Year Plan for the Protection and Development of the Disabled，The “Preschool rehabilitation education development project for disabled children” has been listed as a key project of education for the disabled to accelerate the development of special education in the stage of non- compulsory education.

Action Plan for the Development and Improvement of Preschool Education in the 14th Five Year Plan，Special education courses should be added to the preschool education major in universities, improving the inclusive education ability of normal students.

The introduction of various policy documents has effectively promoted the practice of preschool inclusive education. At present, Beijing, Tianjin, Jiangsu, Henan, and other places do better in domestic preschool inclusive education. For example, Beijing requires all districts to add 1–2 inclusive kindergartens in each school district. In 2019, a total of 63 kindergartens had received children with special needs, and the enrollment rate of children with special needs aged 3–6 years had reached 61.6%. Tianjin required that at least 1–2 general schools and 1–2 kindergartens be designated as pilot schools and kindergartens for autism children’s inclusive education by the end of 2020. Several resettlement schemes, including parent–child kindergartens, special education school’s kindergartens (ordinary class, experimental class), inclusive kindergartens, independent and nearby kindergartens (or connecting with ordinary primary schools) have been explored to meet the needs of children with different degrees of autism. Jiangsu Province vigorously promoted preschool inclusive education by building 1,202 pre-school inclusive education resource centers jointly identified by the four departments of education, civil affairs, health, and the Disabled Persons Federation. They relied on the ordinary kindergartens, which realized full coverage of all towns (streets), so that children with disabilities can enter the appropriate kindergartens relatively near their homes. Henan province promotes preschool inclusion education from the provincial level. At the end of 2019, Henan Province selected five batches of 182 pilot parks for preschool inclusion education. This year (2022), Henan Province has incorporated new plans and initiatives for preschool inclusive education into the third special education promotion plan and the “14th five year plan” for the development and promotion of preschool education, and selected the sixth batch of inclusive education pilot parks.

The quality of life of children with disabilities in kindergartens has been receiving attention. There is concern, however, regarding whether children with disabilities can participate in the main body of activities, exercise their rights and responsibilities, and have a positive impact on the activities.

The current research focused on the definition of children’s participation, the participation ladder, the attitude of teachers and parents to the participation of disabled children, the participation degree of different types of activities, inclusive education strategies, and the social interaction between ordinary children and autistic children.

### Connotations and steps of children’s participation

The United Nations Convention on *The Rights of the Child* states that children have the right to express their opinions on all matters affecting them. It is to ensure that children with independent abilities have the right to express their views freely on all matters affecting them, and adults’ views on children should be treated appropriately according to their age and maturity. Save the Children’s international coalition’s working definition of child participation is that children freely and voluntarily participate in the expression of views, decision-making, or implementation of actions to realize their own or other children’s interests and ensure the realization and protection of their rights. Masao Ono, a former UNICEF representative in China, believes that children’s participation means that children and adolescents are actively involved in the decision-making process about their living environment. This requires two conditions: one is to create an environment, such as a children’s forum or children’s committee; second, there needs to be a platform for child empowerment which allows children to participate in things related to themselves and make use of the knowledge, values, and skills of the participatory environment, and supervise the necessary conditions for their participation in the environment where participation is not allowed.

Hart (1992) first proposed the “ladder of participation” in his book “*Children’s Participation - From Symbolism to Civil Rights,*” which divided “children’s participation” into eight steps: manipulation, decoration, superficial (symbolic), inviting adults to make joint decisions (assigning tasks but notifying them in advance), adult consultation and informing children, adults initiating joint decisions with children, children initiating and deciding by themselves, and children initiating and inviting adults to make joint decisions as subjects. In 1989, Harry Shier proposed the path of children’s participation as follows: children are listened to, children are supported in expressing their opinions, children’s opinions are valued, children participate in the decision-making process, and children share power and responsibility in decision-making. Fanlin et al. (2006) believe that “children’s participation” is divided into five stages: Adults decide and assign children to participate in activities; Children comment on the activities they participate in, but adults do not adopt their suggestions; Children comment on the activities they participate in and their suggestions are adopted by adults; Children propose activities and make decisions with adults; Children organize activities by themselves and invite adults to participate. Chen and Weidong (2006) proposed five levels of children’s participation: adults are fully in charge, adults lead children’s participation, adults and children jointly negotiate, children take the initiative and adults support them, and children are fully in charge. The Lundy (2014) model of Child Participation was established. The model suggests that implementation of Article 12 of the United Nations Convention on the Rights of the Child (CRC) requires consideration of four inter-related concepts: Space, voice, Audience and Influence. Space: Children must be given the opportunity to express a view, Voice: Children must be facilitated to express their views, Audience: The view must be listened to, Influence: The view must be acted upon, as appropriate. Its implementation has generated a sea-change in global understanding of child rights-based participation for both policy and practice.

LaVesser and Berg (2011) compares compared participation patterns and barriers to participation in children with an autism spectrum disorder with those of children with no diagnosis (LaVesser and Berg, 2011). Using the Preschool Activity Card Sort, parent interviews revealed children with an autism diagnosis participate in fewer preschool activities of self-care, community mobility, vigorous leisure, sedentary leisure, social interaction, chores, and education than children with no diagnosis. Njuki analyzed the conceptualization of mild intellectual disability and developmental delay in young children in Sweden, particularly in regard to children’s participation and possible stigmatization in preschool (Njuki, 2013). Phillips and Hogan (2015) explored the association between participation and social competence for preschool aged children with and without disabilities. The Assessment of Preschool Children’s Participation (APCP), a measure of participation in play, skill development, active physical, and social activities of preschool children. Kang et al. (2017) examined the psychometric properties of the Chinese version of the Assessment of Preschool Children’s Participation. Hoydal (2016) finded that natural strongholds for transfers, good meeting points and areas for unsupervised play especially promote participation and play for children with disability.

### Special education in preschool inclusive education needs the basic situation and evaluation tools of children’s participation in activities

Weiya (2011) conducted a questionnaire survey on front-line teachers of ordinary kindergartens in Zhejiang Province and found that children with development defects and children with possible development defects had the highest participation in routine activities in kindergartens, had great differences in participation in corner activities, and had similar participation in structured collective teaching activities, sports activities and games. Among these activities, participation in sports activities was relatively low, with more than half of the two types of children not able to participate into sports activities (Weiya, 2011). Shuangquan et al. (2022) conducted research from the perspective of social participation and found that the social participation of children with special needs was generally worrying, as reflected in the overall low level of peer acceptance and the high level of negative interaction. They also found that girls were more likely to be accepted by peers than boys.

In The Joint Statement, the two authorities of the Division for Early Childhood (DEC) and the National Association for the Education of Young Children (NAEYC) pointed out that high-quality inclusive preschool education should have three basic characteristics, namely accessibility, participation, and support. It is a widely accepted evaluation standard of the International Preschool Inclusive Education Institute (Inclusion, 2009). In 2017, the European Agency for the Development of Special and Inclusive Education cooperated with Education Departments at all levels in 32 European countries to put forward the “ecosystem model of preschool inclusive education,” which described in detail the structure, process, and outcome dimensions of high-quality preschool inclusive education (Wang and Deng, 2022).

At present, the most common tools for the quality evaluation of preschool inclusion education in the world are: Manchester Inclusive Standard, Early Childhood Environment Rating Scale Revised, and Quality of Inclusive Experiences Measure-Revised. Manchester Inclusive Standard examines the quality of inclusive education from three aspects: presence, participation, and achievement. “Presence” refers to the application of enrollment, participation in teaching, and exclusion and withdrawal from the class. “Participation” involves the quality of learners’ educational experience. “Achievement” refers to the learning outcomes of learners (Shuqiong and Junming, 2008). The Early Childhood Environment Rating Scale revised (ECERS-R) developed by Harris et al. is generally considered to be the best evaluation scale to measure the quality of generalized preschool inclusive education (Guodong et al., 2015). The Quality of Inclusive Experiences Measure-Revised (QUIEM-R) scale was developed by Wolery et al. (2000) and was revised in 2005. It includes seven main items: inclusion educational ideas and objectives, support and attitude towards teachers, accessibility of the physical environment, individualized education plans, participation of children with disabilities, teacher-child interaction, and peer interaction. Zhang Boya used the sub-evaluation form of children with special needs to investigate the participation of children with special needs and found that the participation of children with special needs was not ideal (Wolery et al., 2000). In addition, Irwin, a Canadian scholar, developed The Specialink Early childhood Inclusion Quality Scale, which is mainly used to assess children’s experience in an inclusive educational environment, including a concept assessment scale and a practice assessment scale (Irwin, 2005). The existing evaluation tools pay more attention to the overall environment, influencing factors and results of preschool inclusive education, but pay less attention to the process state of children with disabilities’ participation in activities and the degree of participation in specific activities in preschool inclusive education.

In this study, the “Questionnaire on the Participation of Children with disabilities in Inclusive Kindergartens” focuses on the actual participation process and investment of children with disabilities in the field of inclusive education. By consulting the existing domestic and international relevant research literature and group interviews, the researchers conducted comparative analysis, synthesis and refinement to determine the dimensions of the questionnaire. The construction of each dimension and the preparation of the project are carried out in strict accordance with the questionnaire preparation procedure. It is hoped that it can provide operable tools for preschool inclusive education teachers to understand and evaluate the participation of “children with disabilities’ activities.”

## Preparation of the questionnaire on the participation of children with disabilities in preschool inclusive education

### Method

#### Sample

The research recruited kindergarten teachers with inclusive education experience in 10 provinces and 20 cities in China such as Jiangsu, Zhejiang, Anhui, Jilin, Inner Mongolia, Sichuan, Guizhou, Guangxi, Yunnan, and Xinjiang (see Table 1).

**Table 1:** Demographic characteristics.

	Demographic characteristics	*N*	%
Geographical area	Eastern region	160	33.7%
	Central region	152	32.0%
	Western region	163	34.3%
Kindergarten types	Public kindergarten	243	51.2%
	private kindergarten	232	48.8%
Educational background	Junior secondary or below	170	35.8%
	High school	182	38.3%
	University or above	123	25.9%

#### The questionnaire content and scores

The questionnaire items includes the specific environment, social support and participation of children with disabilities in the daily life, games, and collective activities and their compliance with rules in general kindergarten. The survey uses a 5-point Likert scale, and includes 39 items. Each item has five responses, namely, “very consistent,” “conforming,” “inaccurate,” “not conforming,” “totally inconsistent,” recorded as 5, 4, 3, 2, and 1 points, respectively.

The questionnaire is filled in the form of voluntary principle and online. The instruction on the home page of the questionnaire states that this questionnaire is completely anonymous, only used for academic purposes and not for other purposes. Teachers can choose whether to continue to fill it in. Due to the particularity of the tested samples, the convenience sampling method was adopted; that is, accessible and cooperative tested samples were selected. According to the principle of questionnaire preparation, the number of subjects should be 5–10 times the number of items. The total number of questionnaire items was 39. Therefore, 520 questionnaires were issued, and 500 were recovered, giving a recovery rate of 96%. Of the 500, 25 questionnaires with missing or wrong filling were eliminated, meaning that 475 valid data were finally obtained. These data were randomly divided into two parts, one part used for exploratory factor analysis (EFA), and the other for confirmatory factor analysis (CFA).

### Project analysis

Project analysis is a method to analyze the items of a questionnaire according to the results of the test, and to evaluate and screen the better items, so as to further improve the reliability and validity of the questionnaire.

In this study, the project was analyzed using the method of topic and total score correlation. The larger the correlation coefficient is, the higher the degree of distinction is. First, the total score of the questionnaire was calculated, and then the score of each item and the total score were analyzed by Pearson correlation. If the correlation coefficient was less than 0.4, the item would be eliminated (Comrey and Lee, 2013). After analysis, three items were not in conformity with the requirements, so they were deleted in the follow-up study. The specific topics were: 2 (0.226), 15 (0.125), 3 (0.194). The results are shown in Table 2.

**Table 2:** Analysis of the correlation between the item and the total score.

Item no	*r*	*p*	Project no	*r*	*p*	Project no	*r*	*p*
1	0.727	0.000	14	0.723	0.000	27	0.766	0.000
2	0.226	0.000	15	0.125	0.050	28	0.645	0.000
3	0.683	0.000	16	0.194	0.002	29	0.64	0.000
4	0.706	0.000	17	0.753	0.000	30	0.65	0.000
5	0.742	0.000	18	0.75	0.000	31	0.713	0.000
6	0.76	0.000	19	0.772	0.000	32	0.6	0.000
7	0.774	0.000	20	0.769	0.000	33	0.547	0.000
8	0.774	0.000	21	0.772	0.000	34	0.634	0.000
9	0.749	0.000	22	0.662	0.000	35	0.76	0.000
10	0.773	0.000	23	0.763	0.000	36	0.667	0.000
11	0.734	0.000	24	0.807	0.000	37	0.788	0.000
12	0.746	0.000	25	0.811	0.000	38	0.741	0.000
13	0.755	0.000	26	0.771	0.000	39	0.721	0.000

## Data processing of the questionnaire on participation degree of children with disabilities in preschool inclusion education

### Exploratory factor analysis

After the project analysis, the questionnaire included 36 items, which were further explored using factor analysis (EFA). Firstly, in the test results of the fitness of factor analysis: KMO = 0.928, which was greater than 0.6. It is in line with the viewpoint of Kaiser (1974) that the KMO value should be more than 0.6 before the factor analysis can be continued. The sphericity test reached a significant level (*χ*^2^ = 5411.127，df = 153，*p* < 0.001), which indicates that the variables in this study were very suitable for factor analysis.

**Figure 1:**
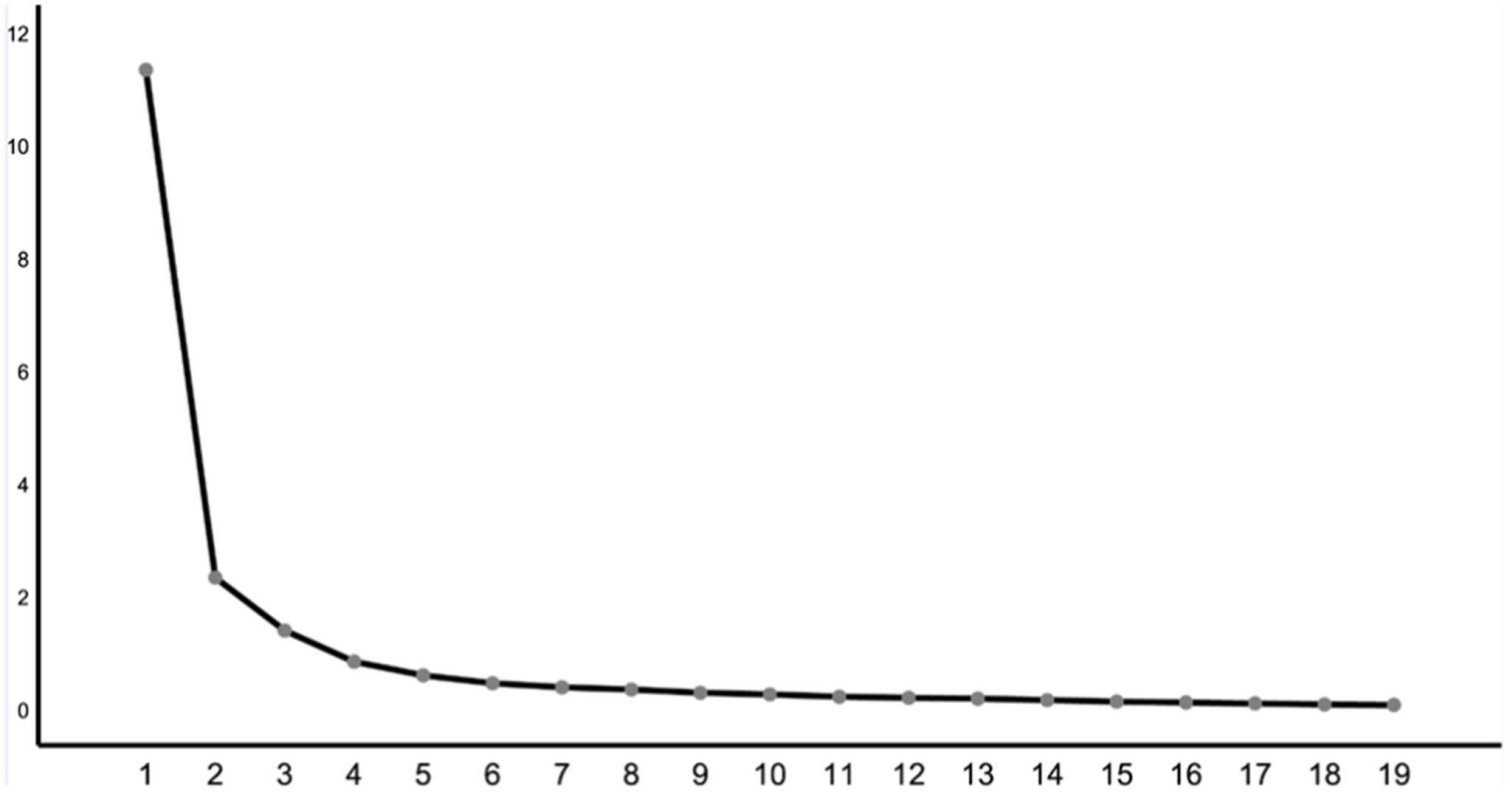
Gravel diagram.

Then, the number of factors was determined by principal component analysis, combining the gravel map (Figure 1), Kaiser criterion, and variance interpretation standard (Kaiser, 1958, 1974). We deleted the items with multiple loads in the way of orthogonal rotation (that is, two or more factor loads are higher than 0.4, and the difference between loads is less than 0.2). According to the standard, 18 items such as 1, 3, 7, 9, and 10 were deleted, and finally a questionnaire on the participation of children with disabilities in preschool inclusive education with 18 items was formed. There were four factors in total, and the interpretation rate of cumulative variance was 85.560%. The results are shown in Table 3.

**Table 3:** Exploratory factor analysis.

Subject	Factor one	Factor two	Factor three	Factor four	Commonality
T9	0.911				0.940
T14	0.910				0.921
T13	0.908				0.946
T12	0.901				0.920
T11	0.901				0.921
T36		0.848			0.794
T32		0.844			0.772
T35		0.785			0.798
T30		0.776			0.790
T38		0.770			0.749
T4			0.857		0.906
T5			0.844		0.932
T6			0.821		0.911
T18			0.711		0.772
T42				0.795	0.805
T40				0.793	0.848
T21				0.784	0.850
T23				0.781	0.828
characteristic value	9.822	3.205	1.284	1.089	
Cumulative contribution rate (%)	54.569	72.374	79.508	85.560	

The four factors are teacher support, learning participation, parents’ attitude, and life participation. Factor 1 includes items 9, 14, 13, 12, and 11. These items describe teachers’ praise and response to children with disabilities, as well as teachers’ attention to and support for social interaction between children with disabilities and ordinary children. Therefore, this factor was named “teacher support.”

Factor 2 includes items 36, 32, 35, 30, and 38. These items describe the cognitive, emotional, and behavioral investment of children with disabilities in learning activities and describe whether children with disabilities can break through shallow and superficial learning and have creative performance. Therefore, this factor was named “learning participation.”

Factor 3 includes items 4, 5, 6, and 18. These items describe the attitude and cognition of ordinary children’s parents in inclusive kindergartens towards preschool inclusive education, as well as the actual support for preschool inclusive education. Therefore, this factor was named “parents’ attitude.”

Factor 4 includes items 42, 40, 21, and 23. These items describe the self-management, rule-abiding, social adaptability, and relationship with others of children with disabilities, so it was named “life participation.”

### Confirmatory factor analysis

**Figure 2:**
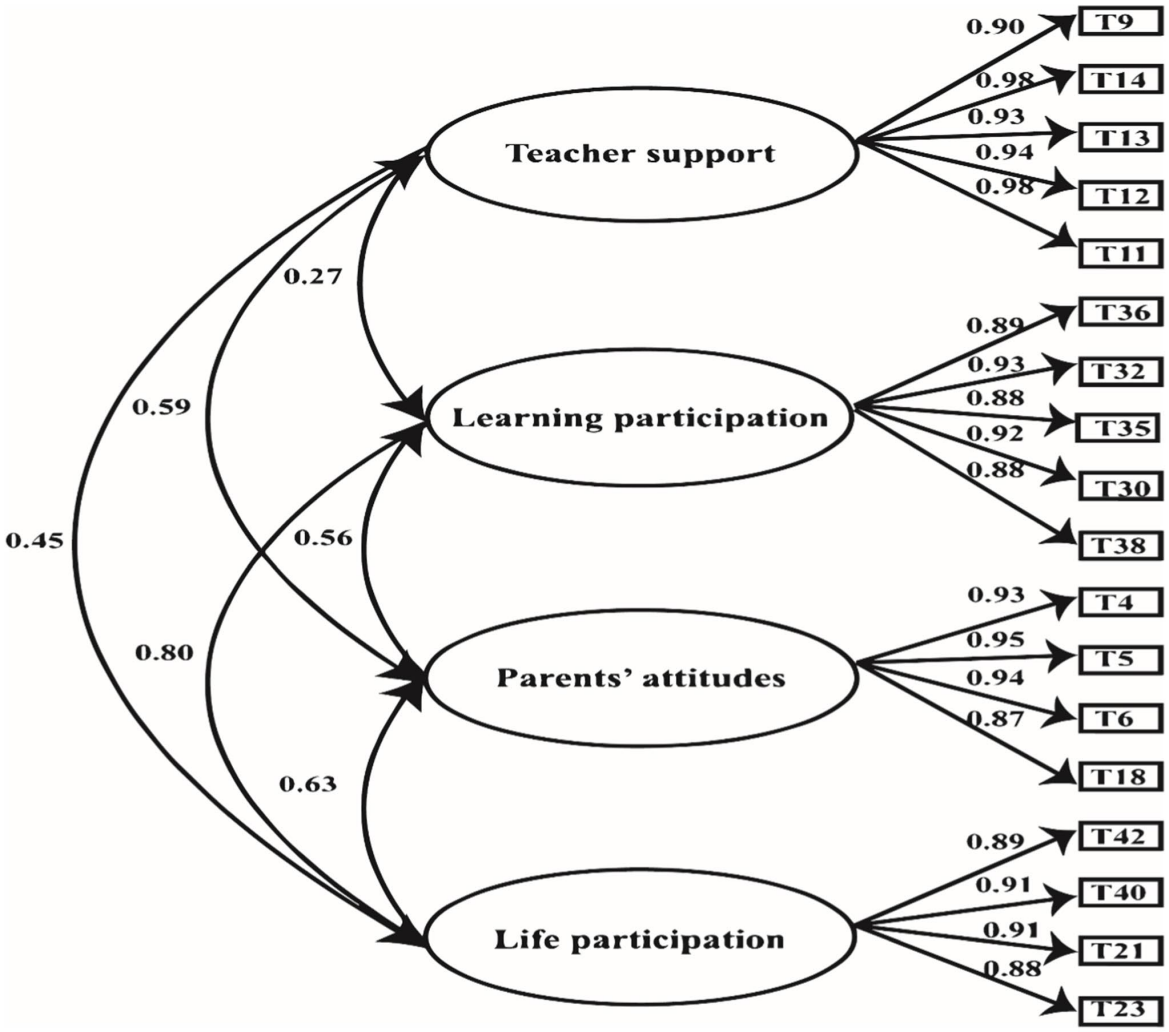
Four Factor model diagram.

We used the Mplus8.3 software to perform confirmatory factor analysis (CFA) on the four-factor model (see Figure 2). The model fitting index is shown in Table 4. It can be seen that all fitting indexes met the following requirements: *χ*^2^/df < 5, Comparative Fit Index ≥ 0.9, Tucker-Lewis Index ≥ 0.9, Root Mean Square Error of Approximation < 0.1, Root Mean Square Error of Approximation < 0.1 (Zhonglin et al., 2004). The standardized load of the four sub-dimensions was between 0.88 and 0.95; the load reached the significant level of 0.01, and the interpretation rate presented a medium to high level (the interpretation rate was greater than 70%), so the model fit well.

**Table 4:** Fitting index of the model.

Fitting index	*χ*^2^/df	CFI	TLI	RMSEA	SRMR
Judgment criteria	<5	≥0.9	≥0.9	<0.1	<0.1
Inspection results	2.573	0.965	0.958	0.083	0.035

CFI, Comparative fit index; TLI, Tucker-lewis index; RMSEA, Root mean square error of approximation; SRMR, Standardized root mean square residual.

### Questionnaire reliability

Reliability is a sign of the reliability or consistency of a questionnaire; that is, the “questionnaire on the participation of children with disabilities in preschool inclusive education” can stably and consistently measure the participation of children with disabilities in activities. Cronbach’s *α* coefficient and split half reliability were used as indicators to test the reliability analysis of the questionnaire. The value range of reliability was between 0 and 1. The closer the reliability coefficient is to 1, the higher the internal consistency of each item of the questionnaire, indicating that the reliability of the questionnaire is better. In the field of Social Sciences, the reliability of a questionnaire above 0.6 is acceptable, and above 0.8 means it has good reliability. The results are shown in Table 5. The Cronbach’s α of the total dimensions of the questionnaire was 0.950, while the Cronbach’s α coefficient of each sub-dimension was between 0.916 and 0.981. The split half reliability of the total dimension of the questionnaire was 0.880. The split half reliability of each sub-dimension was between 0.880 and 0.940. The results show that the reliability of the Activity Participation Questionnaire for Children with disabilities in Preschool Inclusive Education is ideal.

**Table 5:** Reliability analysis.

	Teacher support	Learning participation	Parents’ attitudes	Life participation	Total questionnaire
Cronbach’s α	0.981	0.916	0.952	0.929	0.950
Split half reliability	0.936	0.880	0.940	0.939	0.880
Number of items	5	5	4	4	18

### Questionnaire validity

#### Content validity

Content validity refers to the degree to which the questionnaire or scale items can represent the measured content or behavior goal, that is, the consistency between the measured content and the predicted content goal. The most common evaluation method of content validity is expert evaluation (Fitzpatrick, 1983). Using the “Delphi Method,” this study invited relevant experts and scholars in this field (including many Doctors of education, principals of inclusive education kindergartens, and front-line kindergarten teachers with special education backgrounds) to review and modify the content of the questionnaire. The discussion content included: whether the content presented in the questionnaire can reflect the activity participation of children with disabilities; whether the expression of the questionnaire items was appropriate; and whether the content of the topic was consistent with the actual teaching situation of inclusive kindergarten. After many discussions and many small sample tests, a formal questionnaire was finally formed. The KMO value of the questionnaire was 0.928, and the spherical test result was significant, meaning that it was suitable for exploratory factor analysis. Therefore, all questions had good validity. To sum up, the self-made “Questionnaire on Children with disabilities’ Activity Participation in Inclusive Kindergartens” can truly reflect the activity participation of children with disabilities to a certain extent and has good content validity.

#### Structural validity

The test of structural validity uses the method of Pearson correlation analysis to analyze the correlation between each dimension of the questionnaire and between each dimension and the total score of the questionnaire and takes its correlation coefficient as the investigation index. If the total score of the questionnaire is too low, it indicates that the total score of each dimension is not the same. If it is too high, it indicates that there is multicollinearity in the measurement content. Therefore, it is better to maintain a moderate correlation between the dimensions of the questionnaire between 0.1 and 0.6. It is best to maintain a medium to high degree of correlation between each dimension and the total score of the questionnaire with a correlation coefficient of 0.3–0.8 (Tucker and Lewis, 1973). According to the results shown in Table 6, the correlation coefficient between each dimension was 0.315–0.659, and the correlation coefficient between each dimension and the total score was 0.781–0.836. The results of all correlation coefficients were significant, indicating that the self-designed “Questionnaire on Participation of Children with disabilities in Inclusive Kindergartens” has good structural validity.

**Table 6:** Correlation between dimensions of the questionnaire and the total score.

	Teacher support	Learning participation	Parents’ attitudes	Life participation	Total score of questionnaire
Teacher support	1				
Learning participation	0.315^**^	1			
Parents’ attitudes	0.653^**^	0.472^**^	1		
Life participation	0.470^**^	0.659^**^	0.601^**^	1	
Total score of questionnaire	0.781^**^	0.759^*^	0.837^**^	0.836^**^	1

**p < 0.01.

#### Descriptive analysis

**Figure 3:**
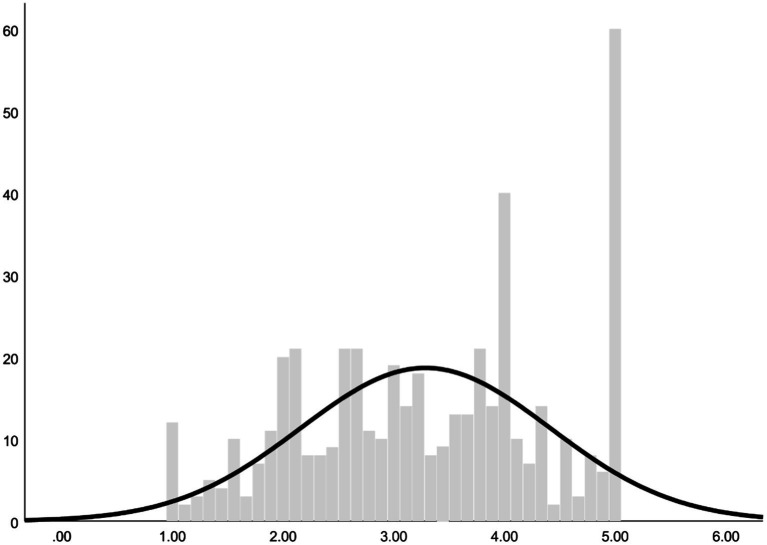
Total score histogram.

On the total score of the questionnaire obtain the following values and include the histogram graph (Tables 7, 8; Figure 3).

**Table 7:** Measures of central tendency and variability.

	Factor 1	Factor 2	Factor 3	Factor 4	Total
Arithmetic mean	3.25	3.26	3.32	3.32	3.29
Median	4.00	3.20	3.25	3.25	3.33
Standard deviation	1.52	1.14	1.23	1.24	1.13

**Table 8:** Characteristics of the distribution of total scores of the questionnaire.

	Value	*p*
Kurtosis	−0.11	>0.05
Asymmetry	−0.95	<0.05
Kolmorov–Smirnov Normality Test	0.07	<0.05

## Conclusion

It can be concluded that the questionnaire on the participation of children with disabilities in preschool inclusive education includes four dimensions: teacher support, parents’ support, learning participation, and life participation, with a total of 18 items. This questionnaire has good reliability and validity and can be used to evaluate the activity participation of children with disabilities in preschool inclusive education. At the same time, it can also be used to investigate the activity participation of ordinary children in inclusive education. Only by starting from all children, paying direct attention to students’ learning results rather than the organizational process, and increasing the participation of all children, can we ensure the quality of inclusive education. The details are shown in Table 9.

**Table 9:** Items of the questionnaire on the participation of children with disabilities in preschool inclusive education.

Teacher support	Q1: Teachers praise children with disabilities
	Q2: Teachers are involved in the conflict between ordinary children and children with disabilities
	Q3: Teachers participate in the social interaction between ordinary children and children with disabilities
	Q4: Teachers support social interaction between ordinary children and children with disabilities
	Q5: Teachers respond to children with disabilities
Parents’ support	Q6: Parents understand inclusive education
	Q7: Parents agree with inclusive education
	Q8: Parents are willing to accept children with disabilities to study in kindergartens
	Q9: Parents participate in inclusive education
Learning participation	Q10: Creative performance
	Q11: Able to speak
	Q12: Imitation of teachers or companions
	Q13: Cooperation with peers
	Q14: Children with disabilities’s needs for peer expression
Life participation	Q15: Can have a quiet nap
	Q16: Observes the rules when eating
	Q17: Walks in an orderly manner with peers
	Q18: Observes the rules when going to the toilet

## Limitations and future research

Although anonymity is declared in the questionnaire stage, kindergarten teachers may still be affected by the “social approval effect,” and may be limited by the limited pre-school inclusion education practice, with fewer samples and insufficient distribution of subjects. In addition, the kindergarten teachers, especially those with a background of inclusion education, may have more burnout. Therefore, there is a regulatory or intermediary effect on the dimensions of this questionnaire. Follow-up research can further explore the relationship between them.

Some scholars have put forward five indicators of inclusive culture in preschool inclusive education; that is, everyone in kindergarten feels very popular, activities in kindergarten are related to children’s personal life, inclusion is the joint effort of everyone related to preschool institutions, all children are expected to achieve goals, and all children are equally valued. We believe that without an inclusive culture, it is impossible to successfully inclusive students with special needs into the mainstream school system, and the inclusive culture of teachers is one of the conditions for the successful inclusion of students with disabilities into the educational organization space (Novachevska et al., 2011; Denisova et al., 2019).

It can be concluded that in the field of preschool inclusive education, due to the influence of the living regional spatial structure of children with special education needs, the subordinate group stratum and the group attitude, the activity participation degree and development level of children with special education needs are different. This requires the joint efforts of the whole society. When inclusive education can really provide effective interaction, fraternity, acceptance and respect for individual differences, the purpose of education for the disabled can not only be written on paper but can also be reflected in the school running concept and live in the actions of teachers and students (Fengming, 2010). This is also one of the directions of preschool inclusive education research in the future.

## Data availability statement

The original contributions presented in the study are included in the article/supplementary material, further inquiries can be directed to the corresponding author.

## Author contributions

LX: design concepts and data collection. ZX: draft the paper and revise it. LZ: retouching the paper. All authors contributed to the article and approved the submitted version.

## Funding

This work was supported by the Key Planning Project of Educational Research (2019), the Chinese Education Society “Research on Activity Participation of Children with Special Needs in Inclusive Kindergarten” (201900652602A).

## Conflict of interest

The authors declare that the research was conducted in the absence of any commercial or financial relationships that could be construed as a potential conflict of interest.

## Publisher’s note

All claims expressed in this article are solely those of the authors and do not necessarily represent those of their affiliated organizations, or those of the publisher, the editors and the reviewers. Any product that may be evaluated in this article, or claim that may be made by its manufacturer, is not guaranteed or endorsed by the publisher.
